# A comparative approach to confirm antibiotic-resistant microbes in the cryosphere

**DOI:** 10.3389/fmicb.2023.1212378

**Published:** 2023-08-03

**Authors:** Daniel Gattinger, Katrin Pichler, Tobias Weil, Birgit Sattler

**Affiliations:** ^1^Institute of Ecology, University of Innsbruck, Innsbruck, Austria; ^2^Research and Innovation Centre, Fondazione Edmund Mach, All'adige, Italy; ^3^Austrian Polar Research Institute, Vienna, Austria

**Keywords:** antibiotic resistance, EUCAST diffusion test, vancomycin, novobiocin, cold habitats, anthropogenic influence

## Abstract

Antibiotic-resistant microbes pose one of the biggest challenges of the current century. While areas with proximity to human impact are closely studied, a lot is yet to learn about antimicrobial resistance in remote regions like the cryosphere. Nowadays, antibiotic (AB) resistance is considered a pollution that has reached the Earth’s most pristine areas. However, monitoring of resistant environmental bacteria therein faces several challenges that inhibit scientific progress in this field. Due to many cultivation-based antibiotic susceptibility tests being optimized for mesophilic pathogenic microorganisms, many researchers opt for expensive molecular biological approaches to detect antibiotic resistance in the cryosphere. However, some disadvantages of these methods prohibit effective comprehensive monitoring of resistant bacteria in pristine areas, hence we suggest established cultivation-based approaches when looking for antimicrobial resistance in the cryosphere. In this study, we compared two common antibiotic susceptibility tests and optimized them to meet the needs of psychrophilic microorganisms. The resulting cultures thereof originated from cryospheric habitats with differing anthropogenic impacts. The results show that these methods are applicable to detect antibiotic resistance in cryospheric habitats and could potentially increase the comparability between studies.

## 1. Introduction

According to the World Health Organization (WHO), antibiotic resistance is one of the biggest global threats of the 21st century. While urbanized areas and hospitals are well-studied, comparably little is known about resistant microbes living in natural or even remote habitats. Although the occurrence of resistant bacteria is assumed to be mainly man-made, recent studies suggest that this perception must be reconsidered after detecting resistance against antibiotics in anthropogenically unaltered environments. Numerous studies indicate that cryospheric habitats like the Arctic (Sjölund et al., 2008) and glaciers (Ushida et al., 2010; Segawa et al., 2013) are no exception and contain a conspicuous number of antibiotic-resistant bacteria and respective resistance genes.

However, genes responsible for resistance against β-lactam, tetracycline, and glycopeptide antibiotics have arisen long before the anthropogenic use of antibacterial substances (D'Costa et al., 2011). This indicates that antibiotic-resistant bacteria in remote areas do not only occur due to the human (mis-)use of antibiotics. Furthermore, so-called intrinsic resistance mechanisms seem to be widely spread in natural habitats and are likely responsible for some antibiotic resistance found in areas with little to no human impact. These general defensive strategies often rely on unspecific efflux pumps and cellular impermeability but also on chromosomally encoded elements (Fajardo et al., 2008; Delcour, 2009; Girgis et al., 2009; Wolff et al., 2009; Alvarez-Ortega et al., 2011) and strategies like extreme slow growth (Gray et al., 2019). This, however, does not mean that antibiotic resistance in anthropogenically unaltered environments like the cryosphere solely occurs naturally. Notably, antibiotic-resistant bacteria can be seen as persistent contaminants in environments that are closely associated with human contact (Kim and Aga, 2007; Zhang et al., 2009), and the translocation of microorganisms carrying antibiotic resistance from areas being strongly impacted by humans to relatively remote habitats is yet to be assessed. The potential dispersal of viable microorganisms through atmospheric transfer (Weil et al., 2017), animal migration (Sjölund et al., 2008), and human mobility (Pavia, 2007; Patel et al., 2018) highlight why the contamination of anthropogenically unaltered environments with antibiotic-resistant bacteria may so far still have been underestimated. The spread of multi-resistance genes like blaNDM-1 into High Arctic soil ecosystems is one of the first to emphasize the extent of the spread of antibiotic resistance in remote areas (McCann et al., 2019).

Regardless of the origin of antibiotic resistance in cryospheric environments, there are still caveats about the potential of psychrophilic and psychrotolerant bacteria to tolerate antibiotics. Especially enhanced melting processes, caused by global warming that ultimately leads to the integration of comparably large abundances of microbes into melting waters, emphasize the importance of understanding antibiotic resistance in cryospheric habitats. Translocation of antibiotic-resistant psychrophilic bacteria and their respective resistance genes through melting processes can potentially lead to a further proliferation of antibiotic resistance mechanisms in urbanized areas due to horizontal gene transfer and vertical inheritance (Allen et al., 2010).

Despite the relatively easy assessment of antibiotic susceptibility in human pathogens with standardized procedures from the European Committee on Antimicrobial Susceptibility Testing (EUCAST) or the Clinical and Laboratory Standards Institute (CLSI), antibiotic resistance research in natural and especially cryospheric habitats lacks standardized methods. This is because mentioned methods, be it the agar disk diffusion test, or the minimum inhibition concentration (MIC) test, are generally optimized for human pathogens which usually prefer different growth conditions than psychrophilic or psychrotolerant bacteria. Hence, methodological adaptations of these tests are required to assess antibiotic resistance in the above-mentioned organisms. The lack of a standardized approach for cultivation-based antibiotic susceptibility tests limits the comparability of these studies. Even though molecular biology addresses part of this problem by looking for antibiotic resistance on a genetic level, culture-based methods still have the advantage of being fast, simple, and at a reasonable price while also detecting unknown phenotypic resistances that are achieved without genetic variations (Corona and Martinez, 2013). Hence, due to their reliability, those methods should be considered when testing cold-loving bacteria.

In this study, we compared the results of two common culture-based antibiotic susceptibility tests: (1) an approach from D'Costa et al. (2006) and Walsh and Duffy (2013) which we will in future refer to as the “cultivation method” and (2) the EUCAST-conform agar disk diffusion method (after Pawlowski et al., 2016). To our knowledge, the cultivation method is the only other cultivation-based antibiotic susceptibility test that is used for environmental samples and non-pathogenic microorganisms. In contrast, the agar-disk diffusion method is a well-established test to determine antibiotic resistance in a clinical environment.

To match the needs of the psychrophilic and psychrotolerant bacteria, both methods were adapted. However, another main goal was to stick to the standardized EUCAST protocol as close as possible. Therefore, the adaptation procedure contained the testing of different incubation temperatures and cultivation media (varying in nutrient composition and agar concentration).

Due to the lack of standardized methods for cryospheric habitats, we propose methods that potentially enhance comparability between future studies. To the best of our knowledge, this is the first comparative study.

## 2. Materials and methods

For our approach, a variety of random cryospheric samples with different levels of anthropogenic influence in the form of high mountain lakes and river water was collected. Ice and snow were collected from various sites across an altitudinal gradient in the Tyrolean Alps.

### 2.1. Sampling sites

**Figure 1:**
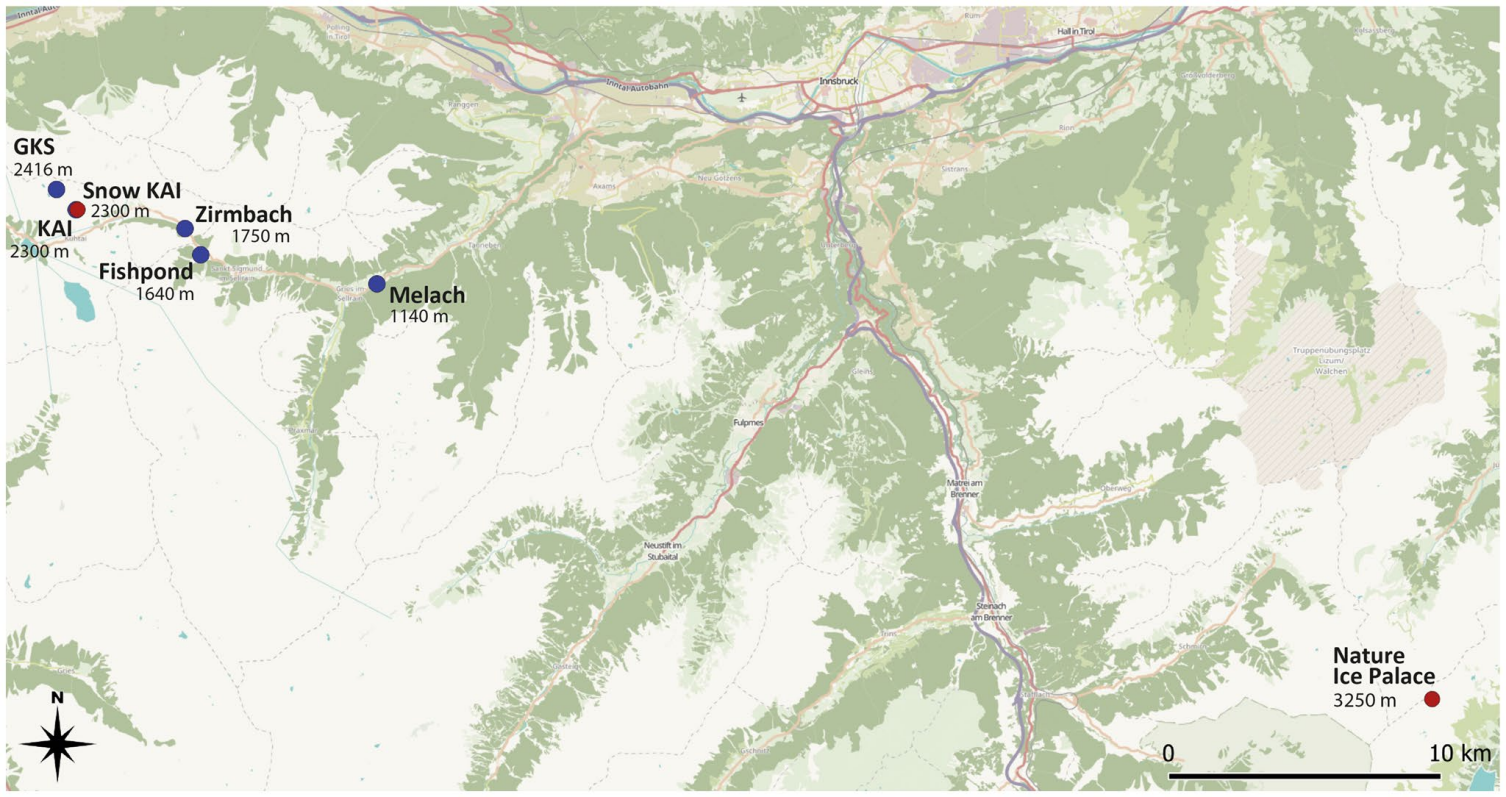
Sampling sites in the Tyrolean alpine space along an altitudinal gradient in the Sellrain valley in the Stubai Alps. The Nature Ice Palace is situated at the Zillertal Alps at 3250 m a.s.l. Ice samples were taken from several locations within the cave. Blue dots indicate water samples, while the red dot highlights the site where snow and ice samples were taken. Source: https://qms.nextgis.com/geoservices/3335.

Water samples were taken in the Sellrain Valley in the Stubai Alps along the watercourse of a mountain river (Zirmbach), a connected fishpond, and an adjacent river (Melach). Zirmbach is a stream in Sellrain with a length of 10 km and originates at an altitude of 1830 m through the confluence of Stockacher-Bach and Klammbach. Its drainage area is around 70 km2 – around the same size as the Melach after the Zirmbach disembogues into it. Lakewater samples have been obtained from Lake Gossenkoelle (GKS) and Lake Kaiser (KAI). Both lakes are located at the Kuehtai skiing resort, with GKS being of natural origin (2.413 m a.s.l.) and KAI a man-made reservoir (2,300 m a.s.l.) to produce technical snow and was built in 2016. Snow samples used in this study have been collected next to KAI. These samples have been collected along an altitudinal gradient (Figure 1).

Ice samples were taken from englacial ice from the “Nature Ice Palace” at the Hintertux Glacier (Figure 1). This touristic walkable crevasse is located at 3250 m a.s.l. and has a constant temperature of 0°C. Ice has hereby been taken from different spots all over the cave, including ice from superficial layers with clear signs of anthropogenic influence (e.g., visible handprints).

With the variety of different sampling sites, we took different levels of anthropogenic influence into account with Hintertux Glacier considered the most affected. Even though it is fairly difficult to evaluate the level of human impact on an absolute level, a relative differentiation of the water samples can be done. Therefore, the water sampling sites can be seen as a transect from most remote lakes (GKS and KAI) to intermediate (Zirmbach) and least remote (Melach). This categorization is mainly based on the geographic proximity to urbanized areas, which is the highest at the River Melach and the lowest at the mountain lakes situated at 2300 m and 2,416 m above sea level, respectively.

The “Nature Ice Palace” located at the Hintertux Glacier is of special interest regarding anthropogenic influence by being a tourist attraction. While again no absolute measure can be determined to define the human impact at this sampling site, numerous daily visitors (50.000 visitors/year, pers. comm. Roman Erler), frequent skin contact, and the lack of good ventilation due to the cave being well-isolated are making these superficial samples presumably one of the most affected by humans within this study.

### 2.2. Sampling

Water was collected into either 250 mL sterile screw-cap bottles or Whirl-Packs®, respectively. Snow was sampled with a cleaned stainless-steel shovel and placed in Whirl-Packs®. For superficial ice, a stainless-steel shovel was used to scratch off ice chips. Cleaning of all sampling devices was done with the respective original sample. Sampling took place between December 2018 and February 2019. After transport to the home institute, snow, and ice were stored at −20°C until further use.

### 2.3. Isolation of pure cultures

To proceed with the antibiotic screening, pure cultures had to be isolated from all sites. Therefore, 1 mL of each snow and water sample was plated on Reasoner’s 2A agar (R2A, Merck) and incubated at 4°C and 20°C, respectively, until visible growth. For incubation, a temperature of 4°C was chosen due to the original cryospheric habitat, while 20°C was applied in parallel to approach the requirements for standardized tests for antibiotic screening despite knowing this would be a compromise toward the tolerability of cold-loving bacteria. Ice and snow samples were processed the same way, except for an initial melting step at 4°C before plating. R2A agar was chosen due to the suitability of cryospheric organisms to grow best on oligotrophic media (Rüthi et al., 2023). Morphological different colonies from all samples were described, transferred into 7 mL of lysogeny broth (LB-media) with a sterile loop, and incubated for 7 days at the respective temperature. LB suits the criteria for the subsequent EUCAST test. After checking their purity with streaking, all isolates were tested for growth at 4, 20, and 37°C. The latter is the standard temperature for antibiotic screening tests, however, not suitable for cryospheric organisms which needed to be confirmed.

### 2.4. Antibiotic susceptibility testing

#### 2.4.1. Antibiotics

We chose two natural antibiotics with different molecular weights to check for diffusion performance: novobiocin and vancomycin. The antibiotic disks were obtained from Oxoid™ (Fisher Scientific). Vancomycin and novobiocin powder (Carl Roth) were dissolved following the manufacturer’s instructions.

#### 2.4.2. Cultivation method

For the first antibiotic susceptibility testing method, the protocol by D'Costa et al. (2006) and Walsh and Duffy (2013) was followed with some minor adjustments. In brief, each cultured bacterium was tested in duplicates for antibiotic susceptibility by plating 100 μL bacterial suspension on R2A agar plates containing 20 μg/mL of one antibiotic. The plates were incubated at the respective temperatures for three days (20°C) or one to two weeks (4°C). A bacterium has been declared resistant if any colony-forming unit was observed. Negative controls included plating of sterile LB (lysogeny broth) media on R2A agar plates containing 20 μg/mL antibiotic. To evaluate the effect of incubation temperature and nutrition media, all cultures with the ability to grow at 4 and 20°C were tested at both temperatures and on R2A and Mueller-Hinton (MH) agar (all nutrition media: Carl Roth). MH agar is set as a standard for the agar disk diffusion method (The European Committee on Antimicrobial Susceptibility Testing, 2021a).

#### 2.4.3. EUCAST agar disk diffusion test

For the agar disk diffusion test, adjustments of the standardized procedure by EUCAST (The European Committee on Antimicrobial Susceptibility Testing, 2021a,b) were needed for psychrophilic and slow-growing organisms. Those adaptations included the previously described reduction of the incubation temperature to 20 and 4°C, respectively, and an increased incubation time for slow-growing cultures of up to 2 weeks. To address one of the major problems when applying the agar disk diffusion test to bacteria from the cryosphere – the lack of breakpoints for non-pathogens – the mean threshold for vancomycin (14.83 mm) was calculated based on all given diameter breakpoints (The European Committee on Antimicrobial Susceptibility Testing, 2021b, see Supplementary Table S3). For novobiocin, which is not included in the EUCAST breakpoint table, thresholds (< 16 mm = resistant) were applied according to the existing literature (Harrington and Gaydos, 1984). Following the EUCAST reading guide, bacteria showing a zone of inhibition that is smaller than the defined breakpoints were considered resistant. The isolates with an inhibition zone diameter bigger or equal to the calculated sensitive breakpoint were considered sensitive to the antibiotic. Apart from these adaptations, we followed the official protocol given by the EUCAST.

To evaluate the effect of nutrition media and incubation temperature on the size of the zone of inhibition, we tested all cultures capable of growing at 4 and 20°C at both temperatures and on R2A and MH.

To examine the reason for potential differences in the size of the zone of inhibition, another experimental design focused on the adjustment of the agar concentration for each growth media. The agar concentration, which is usually 15 g/L in the R2A agar, was adapted to the level of the original MH agar recipe (17 g/L) and the other way around. This resulted in a total of four different agar compositions (hitherto named R2A15, R2A17, MH15, MH17).

### 2.5. Identification of gram (−) bacteria: KOH-test

As vancomycin’s effect is limited on gram-positive bacteria, a gram test was performed beforehand to the actual antibiotic susceptibility test. To determine the cell wall structure, we followed the protocol of the Ryu non staining KOH technique (Ryu, 1938). Only gram-positive isolates were tested for vancomycin resistanceLife Science Identifiers.

### 2.6. 16S sanger sequencing

DNA of all pure cultures was extracted using Qiagen Blood and Tissue DNA Extraction Kit following the recommended procedure of the manufacturer. For the PCR, the universal primers 27F (5’-AGAGTTTGATCCTGGCTCAG-3′) and 1492R (5’-GGTTACCTTGTTACGACTT-3′) were used to amplify the hypervariable regions from V1 to V9 of the 16S rRNA. The PCR reactions were performed in a total volume of 25 μL containing 12.5 μL of 2x PCR MasterMix (Roche), 0.5 μL of each Primer (10 μM), 1 μL of template DNA (50 ng/μL) and 10.5 μL of nuclease-free water. The cycling conditions were as follows: initial denaturation at 95°C for 2 min, followed by 30 cycles of denaturation at 95°C for 30 s, annealing at 60°C for 30 s, elongation at 72°C for 1 min, and final elongation at 72°C for 7 min. Gel electrophoresis was performed to check the correct length of the PCR fragment with a 1 kb + DNA ladder as a reference. Finally, the amplified products were purified using ExoSAP-IT reagent (Thermo Fisher). Sanger sequencing was then performed at the Fondazione Edmund Mach.

Bioinformatics was done in R (4.0.3) and RStudio (1.1.463.0) using the sangeranalyseR package (Chao et al., 2021). The threshold for the quality score was set at 30. Trimmed sequences were blasted against the NCBI database and submitted to the GenBank®.

### 2.7. Statistics

All statistics were performed in R (4.0.3) and RStudio (1.1.463.0) including the following packages: tidyverse, ggpubr, rstatix, networkD3, dplyr, and dabestr. To evaluate differences in the diameter of the zone of inhibition, the dataset was filtered so that cultures that showed zero inhibition in their growth by the antibiotic at any condition (zone of inhibition = 0 mm) were not part of the analysis. If agar plates showed a zone of inhibition on at least one testing condition, the data was included in the statistical calculations. Estimation plots were performed to estimate the paired mean difference between the conditions MH15, MH17, R2A15, and R2A17. The influence of single parameters on the inhibition zone was tested with a Wilcoxon signed-rank test. Differences between the two cultivation-based antibiotic susceptibility tests were evaluated using descriptive statistics.

## 3. Results

### 3.1. Cultivation of isolates from cryospheric samples

In total, we were able to isolate 35 bacterial colonies from all water samples, 21 from ice, and 6 from snow samples. Morphological differences were heeded within but not between sampling sites. Four cultures initially isolated on R2A agar did not grow after transferring them to liquid media, and one culture did not show any sign of growth after the first antibiotic susceptibility test, which is why in total five isolates were excluded from further analysis. Furthermore, no isolate was able to grow at 37°C while all remaining 57 cultures did successfully form colonies on agar plates at 20°C. Seventy percent thereof were able to grow at 4°C. This confirmed the prior assumption that 37°C is not suitable for the growth of cryospheric bacteria. Hence, all further tests were carried out at 20 and 4°C, respectively. All isolates formed colonies on R2A and MH agar. Of the 57 isolates used for further testing, 37 strains were identified as gram-positive bacteria with the KOH test.

### 3.2. Cultivation method

The number of resistant isolates detected with the cultivation method varied between 63 and 83% for novobiocin and 67 to 85% for vancomycin. The biggest variations in the identification of resistant cultures were observed within the different incubation temperatures (Table 1) as in general, more resistances were observed on both nutrition media when the incubation temperature was decreased.

**Table 1:** Antibiotic resistance within all sampling sites with the two different cultivation based antibiotic susceptibility testing strategies performed at different testing conditions with two antibiotics.

Method	Agar	Antibiotic	4°C	20°C
Cultivation method	MH	Novobiocin	80%	63%
		Vancomycin	85%	67%
	R2A	Novobiocin	83%	68%
		Vancomycin	79%	73%
Agar disk diffusion method	MH	Novobiocin	75%	67%
		Vancomycin	77%	64%
	R2A	Novobiocin	75%	54%
		Vancomycin	79%	64%

According to the cultivation method performed at 20°C, most resistances in the water samples were found in the river Melach (75% novobiocin; 80% vancomycin), followed by the fishpond (60%; 88%), Zirmbach (45%; 59%), KAI (17%; 67%) and GKS (17%; 33%). Samples from the ‘Nature Ice Palace’ located at the Hintertux Glacier contained the highest level of resistant isolates (100%; 81%) within all samples. While snow samples collected next to Lake Kaiser had moderate numbers of antibiotic-resistant bacteria (50%; 50%). On average, these numbers were higher in all habitats when the incubation temperature was decreased to 4°C.

### 3.3. EUCAST agar disk diffusion method

On average, 61% of the isolates were resistant to novobiocin and 64% of the gram-positive bacteria were resistant against vancomycin when the agar disk diffusion method was performed at 20°C. These numbers increased to 75% for novobiocin and 78% for vancomycin when the incubation temperature was reduced to 4°C. However, results differed between different incubation conditions such as temperature, nutrition media, and agar concentration. When performed at 20°C, the agar disk diffusion method detected the majority of resistant bacteria within samples from the touristic ice cave at the Hintertux Glacier (98% novobiocin; 81% vancomycin). Among the other sampling sites, Melach contained the highest number of resistant isolates (50%; 50%) followed by the fishpond (50%; 50%), Zirmbach (32%; 64%), and Gossenkoellesee (33%; 33%). The least amount of resistance was observed within the isolates from Kaiser-See (25, 42%) while isolates from snow samples collected next to the Kaiser-See were resistant in 50 and 60% of the cases. The relative number of resistances increased with a decrease in the incubation temperature from 20°C to 4°C in all sampling sites except for the river Zirmbach.

### 3.4. Comparison of two different cultivation-based antibiotic susceptibility testing strategies

**Figure 2:**
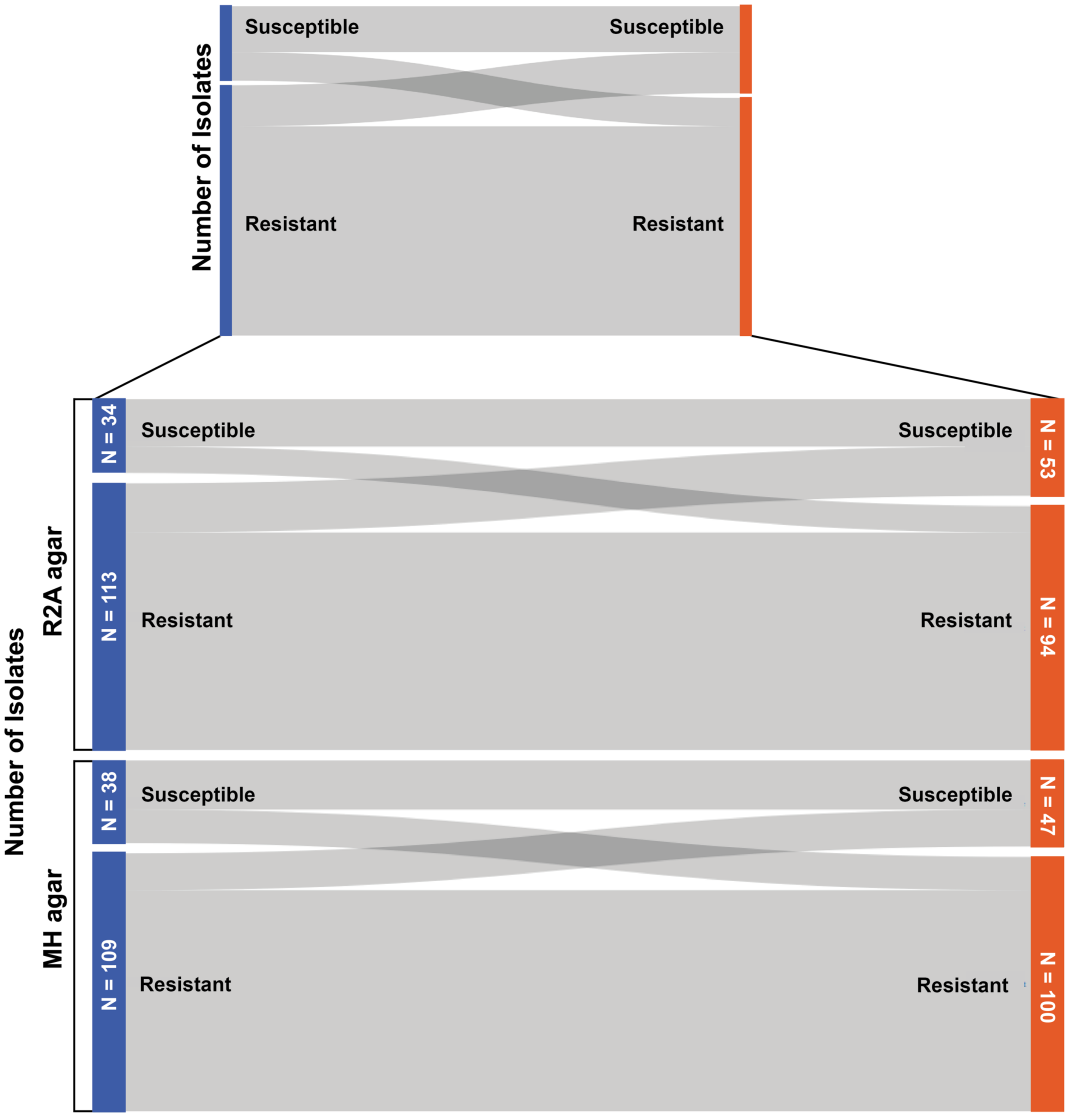
Sankey diagram to display the number of bacteria that changed their resistance status (susceptible/resistant) when tested with the two different antibiotic susceptibility tests.

The two cultivation-based antibiotic susceptibility tests used in this study delivered different results regarding the classification of the bacterial isolates (resistant or sensitive) in approximately 23.8% of the cases. In total, 147 antibiotic susceptibility tests were performed across different nutrition media and testing strategies, including 57 isolates for novobiocin susceptibility tests at 20°C and 40 at 4°C, as well as 31 and 19 gram-positive bacteria being tested for vancomycin resistance at each temperature, respectively. Among these tests, 36.1% of the test results differed when the antibiotic susceptibility tests were performed on R2A agar, and 32% of the test results differed when testing isolates for antibiotic resistance with both testing procedures on MH agar (see Figure 2).

### 3.5. Influence of the agar concentration, incubation temperature, and nutrition media on the size of the inhibition zone

**Figure 3:**
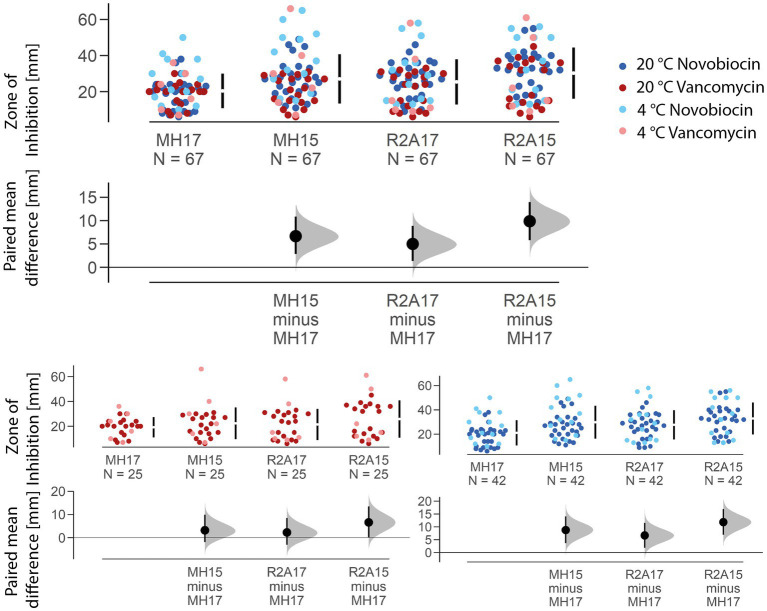
Estimation plot showing the paired mean difference of the zone of inhibition size for the two different nutrition media and agar concentrations used. The mean size of the inhibition zone of MH15, R2A17, and R2A15 is compared to the officially recommended MH agar with an agar concentration of 17 g/L. The biggest mean difference (in total and for each antibiotic) was observed between MH17 and R2A15, resulting in a 9.8758 mm larger zone of inhibition on the Reasoner’s 2A agar.

Compared with the officially recommended MH agar (The European Committee on Antimicrobial Susceptibility Testing, 2021a) with an agar concentration of 17 g/L, all other nutrition media compositions used in this study resulted in increased inhibition zone sizes (Figure 3). The biggest mean difference could be observed between MH17 and R2A15 with 9.87 mm, while MH17 and R2A17 only resulted in a mean difference of 5 mm. On average, the zone of inhibition increased by 6.66 mm on MH15 compared to MH17. The difference in the inhibition zone size caused by the varying agar concentration was significant (*p* < 0.001). Furthermore, different nutrition media (*p* < 0.001) and agar concentrations show significance when using R2A (*p* < 0.001) and MH (*p* < 0.001).

A reduction of the agar concentration from 1.7 to 1.5% resulted in a significant increase of the inhibition zone by 5.17 mm for novobiocin (*p* < 0.001) and 4.36 mm for vancomycin (*p* < 0.01) on R2A agar. Similarly, on MH agar, the inhibition zone increased by 8.74 mm for novobiocin (*p* < 0.001) and 3.18 mm for vancomycin (*p* < 0.05). The largest absolute mean difference was observed between MH17 and R2A15 for novobiocin (+11.8 mm) and vancomycin (+6.6 mm). A significant effect of the incubation temperature could only be seen for novobiocin (*p* < 0.001) but not for vancomycin (*p* = 0.3).

### 3.6. 16S sequencing

Sequences trimmed and controlled for quality had an average length of ~675 bp. No chimeric sequences were found. A total of 15 cultures did not yield satisfactory BLAST results either due to low percentage identity (< 97%) or insufficiently long reads. The Gram status from the KOH test was confirmed in all but six cases. The *Bacillaceae* family was found to be the most prevalent (14) among the isolated colonies with *Pseudomonadacea* following close behind (8) (see Supplementary Table S4).

## 4. Discussion

The emergence of antibiotic resistance has become a major public health concern. With this study, we sought to address the lack of standardized methods for cryospheric communities by comparing different cultivation-based approaches to detect antibiotic resistance in cold habitats.

### 4.1. Cultivation of isolates from cryospheric samples

Out of the initial isolate yield (62), 57 bacterial colonies were successfully grown in liquid media (Luria-Broth) and tested for antibiotic susceptibility.

All cultures used in this study did grow at 20°C whereof 43 cultures had the ability to grow at 4°C, which highlights the psychrophilic character of the isolates usually growing best between 0 and 20°C (Morita, 1975). However, due to limitations in the cultivation of microorganisms from environmental and cold habitats, isolates in this may not be strictly limited to low growing temperatures.

Even though 56% of all bacterial colonies isolated during this study originated from water samples, none were able to grow at 37°C (the recommended temperature for common antibiotic susceptibility tests). This emphasizes the necessity of adaptations for the cultivation-based antibiotic susceptibility tests for bacteria from environmental and especially cryospheric habitats.

### 4.2. Antibiotic resistance and anthropogenic influence

**Figure 4:**
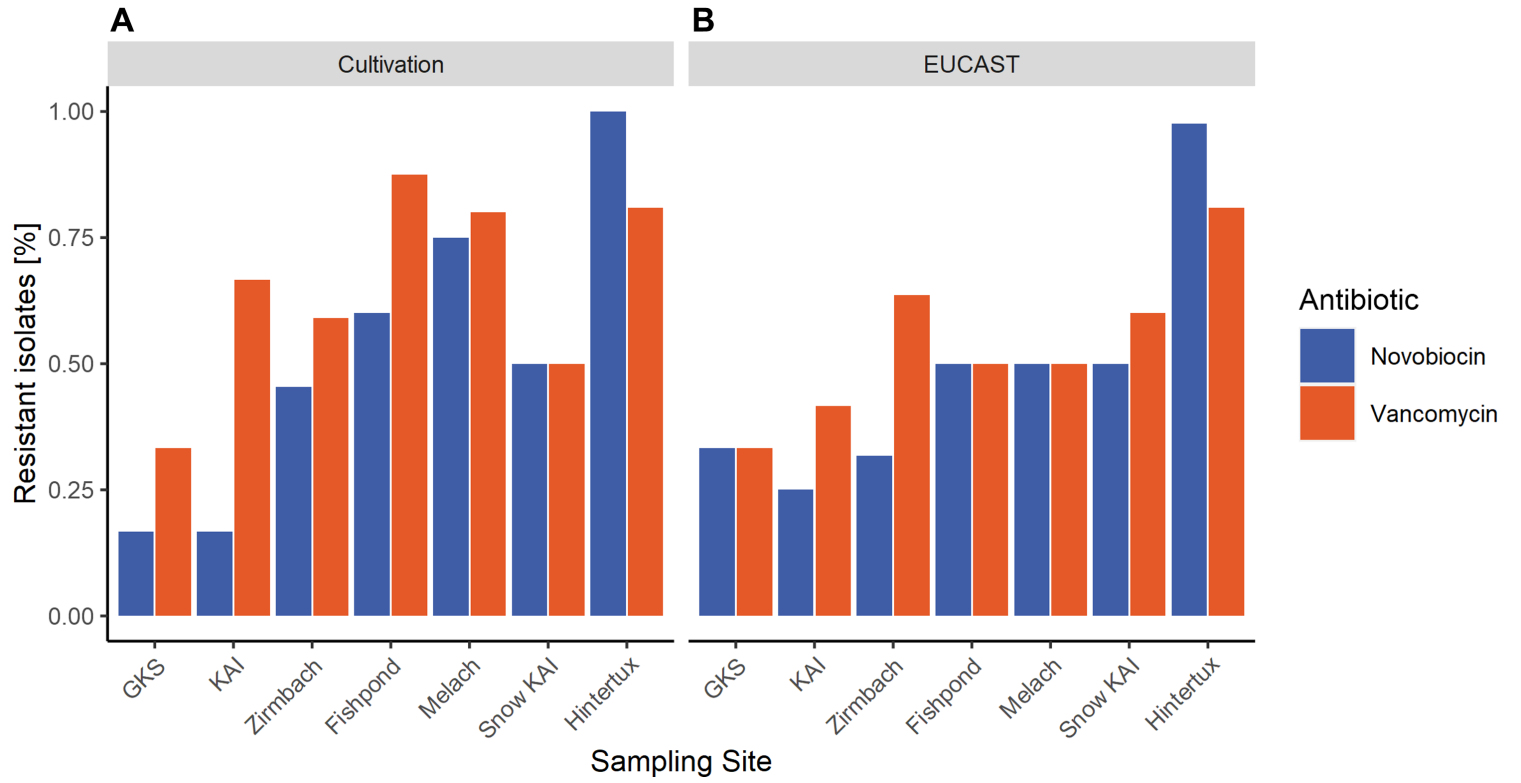
Average percentage of resistant isolates within the sampling sites based on the results of the antibiotic susceptibility tests performed on different nutrition media and incubation temperatures. **(A)** displays the results achieved with the cultivation method at 20°C, while in **(B)** the relative abundance of antibiotic-resistant bacteria detected with the agar-disk-diffusion method at 20°C is mapped.

Antibiotic-resistant microorganisms were found in every sampling site of this study. However, the percentage of resistant bacteria increased along the altitudinal transect from the high alpine lakes (GKS and KAI) to the river Melach, located in a highly populated area. Even though it is difficult to actually measure human impact, an increase in the anthropogenic influence downstream of the water-sample transect can be assumed. On a relative scale, the river Melach, which is showing higher proximity to urbanized areas than any other water sample in this study, is likely to face the highest level of anthropogenic influence. On the other hand, GKS and KAI are only minimally impacted directly by humans, whereas the relative abundance of antibiotic-resistant isolates in these sampling sites is high compared to other little human-impacted areas (Scott et al., 2020). The rural Zirmbach is flowing through some sparsely populated areas and, the observed increase in antibiotic-resistant microbes downstream of the sampled water transect (Figure 4) goes hand in hand with an increased anthropogenic influence due to proximity to humans. These results agree with the general assumption that antibiotic resistance is to a large extent caused by anthropogenic pressure and thus especially present in environments shaped by humans (Kim and Aga, 2007; Berendonk et al., 2015). Furthermore, the same increase in antimicrobial resistance with increased proximity to areas with bigger anthropogenic influence has already been reported before in river networks (McConnell et al., 2018). The effect has been revealed by the selection and the character of the transect but was not the prior scope of this study. The fishpond along the course of the Zirmbach embodies a special case, as it is used for commercial aquaculture. Generally, antibiotics are still widely applied in the fish farming industry across the world and include antibiotics from different classes like penicillins or quinolones (Tuševljak et al., 2012). The use of antimicrobial substances would explain the high numbers of resistance found within the fishpond (Figure 4), even though it is situated outside urbanized areas. However, vancomycin and novobiocin are not approved for antibacterial therapies in aquacultures within the European Union (VO 37/2010 EU), which indicates that these resistances are likely not caused by the use of antibiotic therapies in treating potential fish pathogens.

The river Melach revealed the highest resistances along this transect (Figure 4) which might also be in context with populated areas. In order to cover a high variety of cryospheric habitats for our comparative study, we included an englacial cave open for touristic usage: “Nature Ice Palace” located at the Hintertux Glacier and which is not connected to the transect. The cave is exceptional due to heavy pressure by *ca.* 50.000 yearly visitors and the lack of ventilation. Large numbers of antibiotic-resistant bacteria were found originating from englacial ice. In comparison to other glacial environments around the world, the results indicate that antibiotic resistance does occur in very high numbers within the ice cave (Segawa et al., 2013) (see Figure 4).

### 4.3. Comparison of two different cultivation-based antibiotic susceptibility testing strategies

The comparison of the two different antibiotic susceptibility tests clearly showed that they potentially provide different results regarding the resistance status of bacterial isolates. While it cannot be stated for sure which method is more accurate, the agar disk diffusion method has some clear methodical advantages. It has been tested for years and was already described as an applicable method to evaluate antibiotic resistance in the mid-20th century (Bauer et al., 1959). Apart from the disk-diffusion test being a standardized procedure with an official protocol to follow, it also considers the antibiotic concentration to match its minimum inhibition concentration for each bacterial species – an aspect the cultivation method lacks completely. This absence may be the main reason for the differences between the two methods. The more precise reading guide connected to breakpoints of the agar disk diffusion method – which identifies an isolate as either resistant, susceptible, or (in some cases) intermediate – poses another major cause for differences in the resistance profile of an isolate based on the testing procedure. In total, the two methods delivered different results in ~24% of the treatments (all incubation temperatures and nutrition media). This again highlights the importance of a standardized testing procedure for antibiotic susceptibility testing of environmental and especially cryospheric samples.

### 4.4. Influence of nutrition media, agar concentration, and incubation temperature on the size of the zone of inhibition

In general, an effect on the size of the zone of inhibition has been observed by nutrition media, agar concentration, and incubation temperature.

The effect of the nutrition media on the size of the zone of inhibition is mainly caused by the differences in the agar concentration. A decrease of the agar concentration from 1.7% (original recipe of MH) to 1.5% (original recipe of R2A) ultimately leads to an increased zone of inhibition size at both nutrition media tested. This is likely caused by the enhanced diffusion rate of the antibiotics at a higher diffusion coefficient. According to Fick’s (1855) law the diffusion rate is inversely proportional to the viscosity of the media the molecule has to pass through. The reduction of the agar concentration does decrease the viscosity of the nutrition media, which causes an increased diffusion coefficient and thus an increased diffusion rate for the antibiotics. When comparing R2A15 with R2A17 and MH15 with MH17, respectively, highlights the strong significant effect the agar concentration has on the size of the zone of inhibition (Figure 3).

While other factors like starch may also influence the diffusion rate of antimicrobial substances, these seem to have a much lower impact. However, the differences in the nutrition media compositions might explain the discrepancy between MH15 and R2A15 or MH17 and R2A17 (Figure 3). The significance of the differences indicates that the agar concentration itself is not the only factor that influences the diffusion rate of the antibiotics. Especially, the increased starch concentration of MH agar potentially impacts how easily molecules can diffuse through the agar. Nonetheless, the size of the inhibition zone differed the least when MH17 was compared with R2A17 for both novobiocin (+6.64 mm) and vancomycin (+2.24 mm). Overall, these results suggest that the use of different nutrition media and especially of a different agar concentration can lead to a change in inhibition zone size and, therefore, to a potential misinterpretation of the antibiotic susceptibility of an isolate. Using R2A agar (with 1.5% agar concentration) instead of the officially recommended MH agar (with 1.7% agar concentration) resulted in an average increase of the inhibition zone by 11.8 mm for novobiocin and 6.6 mm for vancomycin, respectively. These differences caused by different nutrition media and agar concentrations can have a drastic impact on the final result of the antibiotic susceptibility test. This is especially relevant because these increases equal roughly 74% (novobiocin) and 44% (vancomycin) of the thresholds given (according to the EUCAST breakpoint table, The European Committee on Antimicrobial Susceptibility Testing, 2021b) to identify a bacterium as either resistant or susceptible to the antibiotic and could therefore lead to a misinterpretation of the actual susceptibility.

The different increase in the zone of inhibition size between the two antibiotics can potentially be explained by the differences in their molecular weight (novobiocin 612.6 g/mol, vancomycin 1449.3 g/mol) as it is again inversely proportional to the diffusion coefficient. According to Fick’s law, diffusion decreases with an increasing radius of the diffusing molecule. Therefore, the diffusion rate is expectedly higher for novobiocin than for vancomycin, which is also represented in the results (Figure 3).

Previous studies described an effect of a decreased incubation temperature and the consequent prolonged incubation period on the results of the agar disk diffusion (e.g., Smith and Kronvall, 2015; Smith et al., 2018) which has also been observed in this study. However, these experiments are usually performed on mesophilic bacteria, while, to the best of our knowledge, nothing is yet reported for psychrophilic and psychrotolerant microorganisms. Our results show that a decrease in the incubation temperature from 20°C to 4°C can cause an increase in the zone of inhibition diameter size when the agar disk diffusion method is applied to psychrophilic and psychrotolerant bacteria. This result is contrary to the expected decreased diffusion rate of molecules at lower temperatures. However, this effect seems to be irrelevant due to the prolonged incubation period of up to 2 weeks for cultures to visibly appear. Interestingly, the significant difference in the size of the zone of inhibition at the two temperatures of interest could only be observed when the antibiotic susceptibility test was performed with novobiocin – a molecule that needs to enter the bacterial cell to inhibit its growth by inhibiting the DNA and/or RNA synthesis (Smith and Davis, 1967). In general, the transport of molecules into bacteria can be mediated by either passive diffusion, facilitated diffusion, or active transport through energy-dependent systems (Chopra, 1989). However, passive diffusion of hydrophilic antibiotics through the cytoplasmic membrane is limited to a size of a maximum of 100 g/mol (Franklin and Leive, 1973). Nevertheless, it is known that even though passive diffusion rates across membranes are lower at cold temperatures, bacterial cells up-regulate membrane transport proteins (de Maayer et al., 2014). This could impact the effective novobiocin uptake at lower temperature levels and explain the average increase in the inhibition zone size. However, even though the zone of inhibition increased significantly when isolates were tested for novobiocin susceptibility at 4°C, the relative number of bacteria determined as resistant did not change. No significant change in the size of the zone of inhibition was observed when the agar disk diffusion method was performed with vancomycin at 20°C and 4°C.

## 5. Conclusion

Overall, our results suggest that the standardized agar disk diffusion test with the recommended nutrition media (MH with an agar concentration of 17 g/L) is well-suited for the analysis of the culturable cryospheric antibiotic susceptibility. The advantage of the established test is not only the standardized protocol and guidelines provided by the EUCAST but also years of research and experience that enhanced the robustness thereof. In addition, the agar disk diffusion test considers the minimum inhibitory concentration and, therefore, relies on the usage of specific antibiotic concentrations and respective breakpoints. However, the latter is species-dependent and only given for common human pathogens, which complicates the application of this method for non-pathogenic environmental bacteria. As the breakpoints within the provided reading table (The European Committee on Antimicrobial Susceptibility Testing, 2021b) do only differ slightly between different bacterial strains, we suggest that using the mean of these thresholds is an appropriate procedure to evaluate antibiotic susceptibility of bacterial strains that are not listed.

The EUCAST protocol for the agar disk diffusion method is optimized for mesophilic pathogenic bacteria and can therefore not be followed regarding incubation temperature and time. This presents another challenge that needs to be tackled to evaluate the antibiotic susceptibility of culturable isolates from environmental and especially cryospheric samples. Sticking as close as possible to the original protocol ensures high-quality standards and is, therefore, strongly recommended. Adaptations regarding the incubation temperature to meet the optimal needs of the bacteria potentially influence the outcome of the results, which is why we suggest using temperatures as close as possible to the official EUCAST guidelines. Apart from that, the nutrition media seem to be an important factor when evaluating the zone of inhibition. Hence, growth on MH agar is strongly advised based on our results. If any other nutrition media is chosen for the testing procedure (e.g., to match the bacterial growing conditions), the adaptation of the agar concentration to 17 g/L might be able to reduce variance in inhibition zone size to a minimum. However, this would need further testing and the most reliable results are to be expected by the use of the suggested nutrition media.

All in all, we suggest that antibiotic susceptibility testing with an adapted version (in terms of incubation temperature and time) of the standardized agar disk diffusion method is applicable to evaluate the culturable resistome of cryospheric habitats and could further enhance the comparability between studies. This method seems especially interesting when complemented with antibiotic resistance gene screening to also assess phenotypic and unknown resistance mechanisms.

## Data availability statement

The original contributions presented in the study are included in the article/Supplementary material, further inquiries can be directed to the corresponding author.

## Author contributions

DG, BS, and TW designed the study. DG and BS collected the samples. DG and KP conducted lab work and evaluation of data (DG: main contributor). DG and BS wrote the manuscript (DG: main contributor) that was reviewed by all DG, BS, KP, and TW. All authors contributed to the article and approved the submitted version.

## Funding

Funding derived by Colonel (IL) J. N. Pritzker, IL ARNG (retired), of the Tawani Foundation by the projects BIO.CRYO and ANTHROPO.SNOW. DG was financed by the University of Innsbruck by a Doctoral Scholarship for 12 months. TW was supported by the Autonomous Province of Trento (Accordo di Programma P2211003I).

## Conflict of interest

The authors declare that the research was conducted in the absence of any commercial or financial relationships that could be construed as a potential conflict of interest.

## Publisher’s note

All claims expressed in this article are solely those of the authors and do not necessarily represent those of their affiliated organizations, or those of the publisher, the editors and the reviewers. Any product that may be evaluated in this article, or claim that may be made by its manufacturer, is not guaranteed or endorsed by the publisher.
