# Supplementary material for: A comparative approach to confirm antibiotic-resistant microbes in the cryosphere
Source: Front Microbiol. 2023 Aug 3;14:1212378. doi: 10.3389/fmicb.2023.1212378 (PMC10435281; doi:10.3389/fmicb.2023.1212378)
Supplement: Supplementary file 1 [file Data_Sheet_1.docx]

Supplementary Material

A comparative approach to confirm antibiotic-resistant microbes in the cryosphere

Daniel Gattinger, Katrin Pichler, Tobias Weil, Birgit Sattler

**
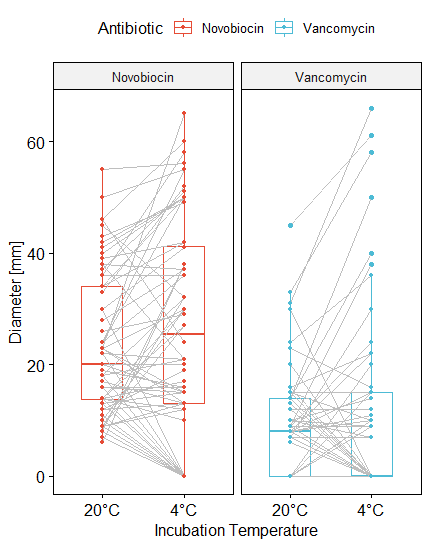
**

**Supplementary Figure 1.** Effect of the incubation temperature on the size of the zone of inhibition for novobiocin and vancomycin.

**Supplementary Table 1.** List of all sampling sites including exact geolocations and sample type. GKS = Gossenkoellesee, KS = Kaiser See, NIP = Nature Ice Palace.

| **Site** | **Sample type** | **GPS coordinates** | **Number of bacterial isolates** |
| --- | --- | --- | --- |
| GKS | Lake/Water | 47°13'45.4"N  11°00'51.4"E | 5 |
| KS | Lake/Water | 47°13'22.7"N  11°01'24.4"E | 7 |
| Snow KS | Snow | 47°13'22.9"N  11°01'26.5"E | 7 |
| Zirmbach | Stream/Water | 47°13'00.5"N  11°04'34.1"E | 11 |
| Fishpond | Fishpond/Water | 47°12'29.3"N  11°05'01.5"E | 5 |
| Melach | Stream/Water | 47°11'54.9"N  11°10'08.5"E | 6 |
| NIP | Glacier/Ice | 47°03'42.4"N  11°40'47.2"E | 21 |

**Supplementary Table 2.** List of the bacterial isolates from all sampling sites including their morphology, gram status and ability to grow at 20 and 4 °C. The gram status has been assessed with the KOH test after Ryu et al. (1938)

|  |  |  |  | **Growth** | |
| --- | --- | --- | --- | --- | --- |
| **Culture Name** | **Sampling Site** | **Morphology** | **Gram status** | **4 °C** | **20 °C** |
| RK 1 | Melach | round, entire, flat, white | positive | - | + |
| RK 2 | Melach | round, entire, flat white | positive | + | + |
| RK 4 | Melach | irregular, filiform, flat, white | negative | - | + |
| RK 5 | GKS | round, filiform, raised, white | positive | - | + |
| RK 6 | GKS | irregular filiform, flat, white | positive | - | + |
| RK 7 | Zirmbach | round, entire, flat, red | negative | - | + |
| RK 8 | Zirmbach | round, entire, flat, yellow | positive | - | + |
| RK 9 | Zirmbach | round, entire, flat, white | positive | - | + |
| RK 10 | Zirmbach | irregular, undulate, flat, yellow | positive | + | + |
| RK 11 | Zirmbach | irregular, filiform, flat, white | positive | - | + |
| RK 12 | KS | round, entire, flat, orange | positive | - | + |
| RK 13 | KS | round, entire, raised, brown | positive | - | + |
| RK 14 | KS | round, entire, flat yellow | positive | + | + |
| RK 15 | KS | round, entire, flat, white | positive | - | + |
| RK 17 | Snow KS | round, entire, flat, yellow | negative | + | + |
| RK 18 | Snow KS | irregular, undulate, flat, white | positive | - | + |
| RK 19 | Snow KS | round, entire, flat, white | negative | - | + |
| RK 20 | Snow KS | irregular, undulate, flat, yellow | negative | - | + |
| RK 21 | Fishpond | round, entire, flat, yellow | positive | - | + |
| RK 22 | Fishpond | round, entire, flat, yellow | positive | - | + |
| RK 23 | Fishpond | round, entire, raised, white | positive | - | + |
| RK 24 | Snow KS | round, entire, fat, yellow | positive | + | + |
| RK 25 | Snow KS | irregular, filiform, flat, yellow | positive | - | - |
| RK26 | Snow KS | round, entire, raised, translucent | positive | + | + |
| RK 27 | Zirmbach | round, entire, raised, white | negative | + | + |
| RK 28 | Zirmbach | round, entire, flat, purple | positive | + | + |
| RK 29 | Zirmbach | irregular, filiform, flat, yellow | negative | + | + |
| RK 30 | Zirmbach | round, entire, raised, yellow | negative | + | + |
| RK 31 | Zirmbach | round, entire, raised, red | positive | + | + |
| RK 32 | Zirmbach | irregular, filiform, flat, white | negative | + | + |
| RK 33 | Fishpond | round, entire, flat, white | negative | + | + |
| RK 34 | Fishpond | round, entire, flat yellow | positive | + | + |
| RK 35 | Melach | round, entire, raised, yellow | positive | + | + |
| RK 36 | Melach | round, entire, raised, white | negative | + | + |
| RK 37 | Melach | irregular, entire, flat, yellow | positive | + | + |
| RK 38 | KS | round, filiform, flat, white | negative | + | + |
| RK 39 | KS | round, entire, convex, yellow | positive | - | - |
| RK 40 | GKS | round, entire, raised, white | positive | - | - |
| RK 41 | KS | round, entire, raised, white | positive | - | - |
| RK 42 | GKS | round, entire, convex, translucent | negative | + | + |
| RK 43 | GKS | round, entire, raised, white | positive | - | - |
| HT 1 | NIP | round, entire, flat, pink | negative | + | + |
| HT 2 | NIP | round, entire, flat, yellow | negative | + | + |
| HT 3 | NIP | round, entire, raised, white | positive | + | + |
| HT 4 | NIP | irregular, undulate, flat, yellow | positive | + | + |
| HT 5 | NIP | round, entire, flat, white | positive | + | + |
| HT 6 | NIP | irregular, entire, flat, yellow | positive | + | + |
| HT 7 | NIP | round, entire, raised, red | positive | + | + |
| HT 8 | NIP | round, undulate, flat, white | negative | + | + |
| HT 9 | NIP | round, entire, flat, yellow | positive | + | + |
| HT 10 | NIP | round, undulate, flat, white | negative | + | + |
| HT 11 | NIP | round, entire, flat, white | positive | + | + |
| HT 12 | NIP | round, entire, flat translucent | positive | + | + |
| HT 13 | NIP | round, undulate, flat, yellow | positive | + | + |
| HT 14 | NIP | round, entire, flat, white | positive | + | + |
| HT 15 | NIP | round, entire, flat, yellow | positive | + | + |
| HT 16 | NIP | round, undulate, flat, translucent | positive | + | + |
| HT 17 | NIP | round, entire, flat, white | negative | + | + |
| HT 18 | NIP | round, entire, raised, white | negative | + | + |
| HT 19 | NIP | round, entire, raised, translucent | negative | + | + |
| HT 20 | NIP | round, translucent, flat, white | negative | + | + |
| HT 21 | NIP | round, entire, raised, white | negative | + | + |

**Supplementary Table 3.** Breakpoint/Size of the zone of inhibition for the agar disk diffusion test based on recommended susceptibility thresholds by the EUCAST (eucast.org 2021b). *Harrington and Gaydos 1984

| **Phyla** | **Antibiotic Family** | **Antibiotic** | **Disk content [µg]** | **S ≥ [mm]** | **R < [mm]** |
| --- | --- | --- | --- | --- | --- |
| *Enterococcus spp.* | Glycopeptides | Vancomycin | 5 | 12 | 12 |
| *Streptococcus groups A, B, C, D* | Glycopeptides | Vancomycin | 5 | 13 | 13 |
| *Streptococcus pneumoniae* | Glycopeptides | Vancomycin | 5 | 16 | 16 |
| *Viridans group streptococci* | Glycopeptides | Vancomycin | 5 | 15 | 15 |
| *Corynebacterium spp.* | Glycopeptides | Vancomycin | 5 | 17 | 17 |
| *Aerococcus sanguinicola/urniae* | Glycopeptides | Vancomycin | 5 | 16 | 16 |
| **Calculated mean value** | | | | **14,83** | **14,83** |
| - | Aminocumarines | Novobiocin | 5 | 16* | 16* |

**Supplementary Table 4.** Results of the 16S rRNA Sanger sequencing. Sequences were submitted to GenBank^®^.

| **Culture Name** | **Family** | **Genus** | **Accession number** |
| --- | --- | --- | --- |
| RK 1 | *Bacillaceae* | *Bacillus* | OR225929 |
| RK 2 | *Bacillaceae* | *Bacillus* | OR225930 |
| RK 4 | *Pseudomonadaceae* | *Pseudomonas* | OR225931 |
| RK 5 | *Bacillaceae* | *Bacillus* | OR225932 |
| RK 6 | *Bacillaceae* | *Bacillus* | OR225933 |
| RK 7 | *Pseudomonadaceae* | *Pseudomonas* | OR225934 |
| RK 8 | *Micrococcaceae* | *Paenarthrobacter* | OR225935 |
| RK 9 | *Bacillaceae* | *Bacillus* | OR225936 |
| RK 10 | *Micrococcaceae* | *Agreia* | OR225937 |
| RK 11 | *-* | *-* |  |
| RK 12 | *Bacillaceae* | *Bacillus* | OR225938 |
| RK 13 | *-* | *-* |  |
| RK 14 | *Microbacteriaceae* | *Plantibacter* | OR225939 |
| RK 15 | *Bacillaceae* | *Bacillus* | OR225940 |
| RK 17 | *Oxalobacteraceae* | *Janthinobacterium* | OR225941 |
| RK 18 | *Bacillaceae* | *Bacillus* | OR225942 |
| RK 19 | *Comamonadaceae* | *Acidovorax* | OR225943 |
| RK 20 | *Rhodobacteraceae* | *Paracoccus* | OR225944 |
| RK 21 | *Flavobacteriaceae* | *Flavobacterium* | OR225945 |
| RK 22 | *Bacillaceae* | *Bacillus* | OR225946 |
| RK 23 | *Bacillaceae* | *Bacillus* | OR225947 |
| RK 24 | *Bacillaceae* | *Lysinibacillus* | OR225948 |
| RK26 | *-* | *-* |  |
| RK 27 | *-* | *-* |  |
| RK 28 | *Flavobacteriaceae* | *Flavobacterium* | OR225949 |
| RK 29 | *-* | *-* |  |
| RK 30 | *Flavobacteriaceae* | *Flavobacterium* | OR225950 |
| RK 31 | *-* | *-* |  |
| RK 32 | *Oxalobacteraceae* | *Massilia* | OR225951 |
| RK 33 | *Flavobacteriaceae* | *Flavobacterium* | OR225952 |
| RK 34 | *-* | *-* |  |
| RK 35 | *Pseudomonadaceae* | *Pseudomonas* | OR225953 |
| RK 36 | *Flavobacteriaceae* | *Flavobacterium* | OR225954 |
| RK 37 | *-* | *-* |  |
| RK 38 | *Oxalobacteraceae* | *Janthinobacterium* | OR225955 |
| RK 42 | *-* | *-* |  |
| HT 1 | *Pseudomonadaceae* | *Pseudomonas* | OR225914 |
| HT 2 | *Sphingomonadaceae* | *Sphingomonas* | OR225915 |
| HT 3 | *-* | *-* |  |
| HT 4 | *Pseudomonadaceae* | *Pseudomonas* | OR225916 |
| HT 5 | *-* | *-* |  |
| HT 6 | *-* | *-* |  |
| HT 7 | *Bacillaceae* | *Bacillus* | OR225917 |
| HT 8 | *Staphylococcaceae* | *Staphylococcus* | OR225918 |
| HT 9 | *Sphingomonadaceae* | *Sphingomonas* | OR225919 |
| HT 10 | *Yersiniaceae* | *Yersinia* | OR225920 |
| HT 11 | *-* | *-* |  |
| HT 12 | *Bacillaceae* | *Bacillus* | OR225921 |
| HT 13 | *Oxalobacteraceae* | *Massilia* | OR225922 |
| HT 14 | *-* | *-* |  |
| HT 15 | *Yersiniaceae* | *Yersinia* | OR225923 |
| HT 16 | *Bacillaceae* | *Bacillus* | OR225924 |
| HT 17 | *Oxalobacteraceae* | *Janthinobacterium* | OR225925 |
| HT 18 | *Pseudomonadaceae* | *Pseudomonas* | OR225926 |
| HT 19 | *Pseudomonadaceae* | *Pseudomonas* | OR225927 |
| HT 20 | *Pseudomonadaceae* | *Pseudomonas* | OR225928 |
| HT 21 | *-* | *-* |  |
